# Bioinformatics design of peptide binding to the human cardiac troponin I (cTnI) in biosensor development for myocardial infarction diagnosis

**DOI:** 10.1371/journal.pone.0305770

**Published:** 2024-10-22

**Authors:** Muhammad Miftah Jauhar, Filasta Rachel Damairetha, Etik Mardliyati, Mokhamad Fakhrul Ulum, Putri Hawa Syaifie, Fahmi Fahmi, Ardianto Satriawan, Wervyan Shalannanda, Isa Anshori

**Affiliations:** 1 COE Life Sciences, Nano Center Indonesia, Jl. PUSPIPTEK, South Tangerang, Banten, Indonesia; 2 Biomedical Engineering, Graduate School of Universitas Gadjah Mada, Sleman Regency, Special Region of Yogyakarta, Indonesia; 3 School of Electrical Engineering and Informatics, Bandung Institute of Technology, Bandung, West Java, Indonesia; 4 Research Center for Vaccine and Drugs, National Research and Innovation Agency (BRIN), Cibinong, West Java, Indonesia; 5 School of Veterinary Medicine and Biomedical Sciences, IPB University (Bogor Agricultural University), Bogor, West Java, Indonesia; 6 Department of Electrical Engineering, Faculty of Engineering, Universitas Sumatera Utara, Medan, North Sumatera, Indonesia; 7 Center for Health and Sports Technology, Bandung Institute of Technology, Bandung, West Java, Indonesia; 8 Research Center for Nanosciences and Nanotechnology (RCNN), Bandung Institute of Technology, Bandung, West Java, Indonesia; ICFAI Foundation for Higher Education Faculty of Science and Technology, INDIA

## Abstract

Cardiovascular disease has reached a mortality rate of 470,000 patients each year. Myocardial infarction accounts for 49.2% of these deaths, and the cTnI protein is a crucial target in diagnosing myocardial infarction. A peptide-based bioreceptor design using a computational approach is a good candidate to be developed for a rapid, effective, and selective detection method for cTnI although it is still lacking in study. Hence, to address the scientific gap, we develop a new candidate peptide for the cTnI biosensor by bioinformatics method and present new computational approaches. The sequential point mutations were made to the selected peptide to increase its stability and affinity for cTnI. Next, molecular docking was performed to select the mutated peptide, and one of the best results was subjected to the molecular dynamics simulation. Finally, the results showed that the best peptide showed the lowest affinity and good stability among other mutated peptide designs for interacting with the cTnI protein. In addition, the peptide has been tested to have a higher specificity towards cTnI than its major isomer, sTnI, through molecular docking and molecular dynamics simulation. Therefore, the peptide is considered a good potential bioreceptor for diagnosing myocardial infarction diseases.

## Introduction

Cardiovascular disease (CVD) remains the most dead-caused leading in the world and contributes to the excess health system costs worldwide. Moreover, the CVD burden is also attributable to 88 risk factors for other diseases [1]. One of the most common cardiovascular diseases is myocardial infarction (MI). Myocardial infarction, generally known as a heart attack, is a disease when the heart muscle or coronary heart is absent from supplying blood or so little supply that the heart cannot sustain its function [2]. Hence, to ease the burden of the disease, early diagnosis is very important to be developed and optimized.

Cardiac troponins (cTns) stand out as highly valuable and specific indicators for cardiovascular conditions, notably acute myocardial infarction. Additionally, these biomarkers have applicability in evaluating the extent of myocardial damage in non-cardiac illnesses that may adversely affect the cells of the cardiac muscle tissue [3]. Presently, the primary approach employed for identifying and measuring troponin involves electrochemiluminescence or an electro-generated chemiluminescence immunoassay (ECLIA), a robust and adaptable detection technique applicable to numerous biomarkers [4, 5]. Despite its utility in troponin detection, ECLIA comes with certain drawbacks, with one notable concern being its considerable expenses. This method is cost-intensive and demands a well-equipped laboratory furnished with advanced instrumentation and skilled personnel.

Some previous research was already conducted either fabricating biosensor instruments/devices or designing the biomolecular recognition elements (BREs) to gain more specific interaction or to get an easier application. Prakash and their team [6, 7], developed a nano biosensor device using nanofiber embedded with SU-8 photoresist to improve the detection of myoglobin, an important biomarker for the onset of Acute Myocardial Infection (AMI). The BREs they used were monoclonal antibody myoglobin. In the next year, they also fabricated the biosensor instrument using a silicon nanowire field effect transistor (SiNW FET) for cardiac troponin I (cTnI) in acute myocardial infarction (AMI) and the cTnI monoclonal antibody (mAb-cTnI) was used as the BREs [8].

On the other side, some research was done to employ the engineering BREs to enhance their specificity in the biosensor field. Small peptides are considered a good candidate for the development of BREs because of their ease of synthesis, low cost, environmental stability, and low molecular weight. Moreover, a lot of peptide sources can be applied to develop as potential BREs and they can interact with various surfaces or can be functionalized to form stable and good biosensor complexes [9, 10],. There was some previous research that developed peptides as BREs for various biosensor diseases using a computational approach. Xiao [10] utilized a computational approach using a functionalized algorithm to develop peptide BREs for the cTnI protein and validated them in the wet lab. Next, Badhe [11] designed peptide biosensing and functionalized them into graphene and carbon nanotube (CNT) as SARS-CoV-2 biosensor development. In the next year, Mastouri [9] developed oligopeptides for capture and reporting probes for interleukin-6 biosensing. From the research, the small-size peptide BRE can reveal higher sensitivity and a lower detection limit compared to the antibody.

A wide array of biorecognition elements are available, spanning from naturally found to artificially engineered structures. BREs in the biosensor field are widely used such as DNA/RNA, peptide, antibody, and molecularly imprinted polymers (MIPs). Furthermore, the development of BREs with a high specificity and selectivity to the biomarker’s target such as the cTnI protein remains a challenge today. Cases of false positives may occur in detecting troponin concentration in the patient’s blood. These cases come because of the less-selectivity of the BREs to detect the desired troponin. They are often associated with other detections such as fibrin clots, heterophile antibodies, alkaline phosphatase, rheumatoid factor, and cross-reactions of diagnostic (anti-cTn) antibodies with troponin molecules released from skeletal muscle [12]. Therefore, to address these challenges requires a rapid, efficient, and automated method for the design of high-affinity BREs.

The peptide development for the cTnI protein biosensor has been developed by some previous research. Park developed short linear binding peptides obtained using phage display [13]. Next, Wang improved the previous peptide from the phage display with a thiol-containing cysteine residue [14]. These peptides are widely used in some research as a base peptide binding-cTnI protein for the next years. Furthermore, Xiao [10] made some peptides for cTnI binding using a computational algorithm based on the peptide from Park [13] as the parental affinity peptide. In our present work, we develop a new peptide binding-cTnI protein using a computational approach different from Xiao [10]. Instead of using a parental peptide from previous research such as Park [13] and Wang [14], we design some new peptides by selecting their interaction with the cTnI protein from the protein database and then optimizing the selected peptide design using the sequential point mutation. Hence, our study reveals the new potential candidate peptide as the cTnI protein biosensor also provides a new computational approach that can aid the optimization of the development of cTnI peptide-based biosensors.

## Materials and methods

### cTnI protein modeling and refinement

cTnI protein is developed by separating its structure from the complex troponin protein structure from the RCSB database (https://www.rcsb.org/) (code PDB ID: 4Y99, chain C). The missing residues from Lys139 until Val147 were modeled using PyMOL software and the ModLoop web server (https://modbase.compbio.ucsf.edu/modloop/). The complete structure was validated using ERRAT and Ramachandran plot assessment in the SAVES6.0 package web server (https://saves.mbi.ucla.edu/).

### Peptide biosensor modeling

To develop a peptide for the cTnI protein biosensor, the important residues from the cTnI protein from the RCSB database were analyzed. To perform this, the FTsite web server (https://ftsite.bu.edu/) [15–17] was used to determine the binding site of the cTnI protein. Furthermore, the important residue analysis was also obtained from previous research to gain a more active site [18]. After determining the binding site, PyMOL was used to select residues that make contact with the binding site residue and within a distance maximum of 4 Å. To build a 3D peptide structure, the trRosetta web server is used (https://yanglab.nankai.edu.cn/trRosetta/). The literature peptide from Park et al. was also modeled as a comparison [13]. Then, similar to the previous step, the 3D structure of the peptides was also validated using ERRAT and Ramachandran plot.

### Molecular docking

To conduct the molecular docking process, ClusPro2.0 (https://cluspro.bu.edu/home.php) and HADDOCK 2.4 web servers (https://wenmr.science.uu.nl/haddock2.4/) were used to ensure the best peptide candidate based on the affinity energy. Both the candidate peptide and the cTnI protein were submitted to the web servers in the PDB format. To obtain the binding affinity energy, the complexes from two web servers were submitted to the PRODIGY web server (https://wenmr.science.uu.nl/prodigy/).

### Molecular interaction analysis

The interaction between peptide and cTnI protein was also analyzed using the PDBSum web server (https://www.ebi.ac.uk/thornton-srv/databases/pdbsum/). The hydrophobic region was also analyzed to support the interaction characteristic of the complex using Discovery Studio Visualizer 2021 software. Then, the conserved region of the cTnI protein structure was analyzed by the MESSA web server (http://prodata.swmed.edu/MESSA/MESSA.cgi) [19].

### Peptide point mutation

After obtaining the best candidate peptide from the molecular docking and interaction analysis, the point mutation approach is subjected to the peptide. the BeAtMuSiC webserver (http://babylone.ulb.ac.be/beatmusic/) was used to conduct this point mutation [20]. The complex of the selected peptide with the cTnI protein was submitted to the server. The mutation process was done by a random mutation in the peptide structure. According to the result of the BeAtMuSiC web server, the peptide was modeled in the 3D structure, validated, and processed to the molecular docking similar to the previous second and third steps to obtain the information of the binding energy affinity and its structure characteristic.

### Molecular dynamics simulation

A molecular dynamics simulation was performed to assess the interaction stability of the peptide-cTnI protein biosensor system complex. GROMACS software was used for the simulation process. The CHARMM36 force field was applied, along with the water TIP3P model. The system complex was placed in a rhombic dodecahedron box, positioned 1.0 nm from the edge. The solvent molecule configuration was set to SPC216 (simple point charge), and chloride ions were added to neutralize the charge. The steepest descent minimization was set to 50.000 steps to minimize the energy system. The system complex was placed in a rhombic dodecahedron box, positioned 1.0 nm from the edge. The solvent molecule configuration was set to SPC216 (simple point charge), and chloride ions were added to neutralize the charge. PyMol was also used to visualize the interaction complex.

## Results

### Bioreceptor and biomarker modelling

**Fig 1:**
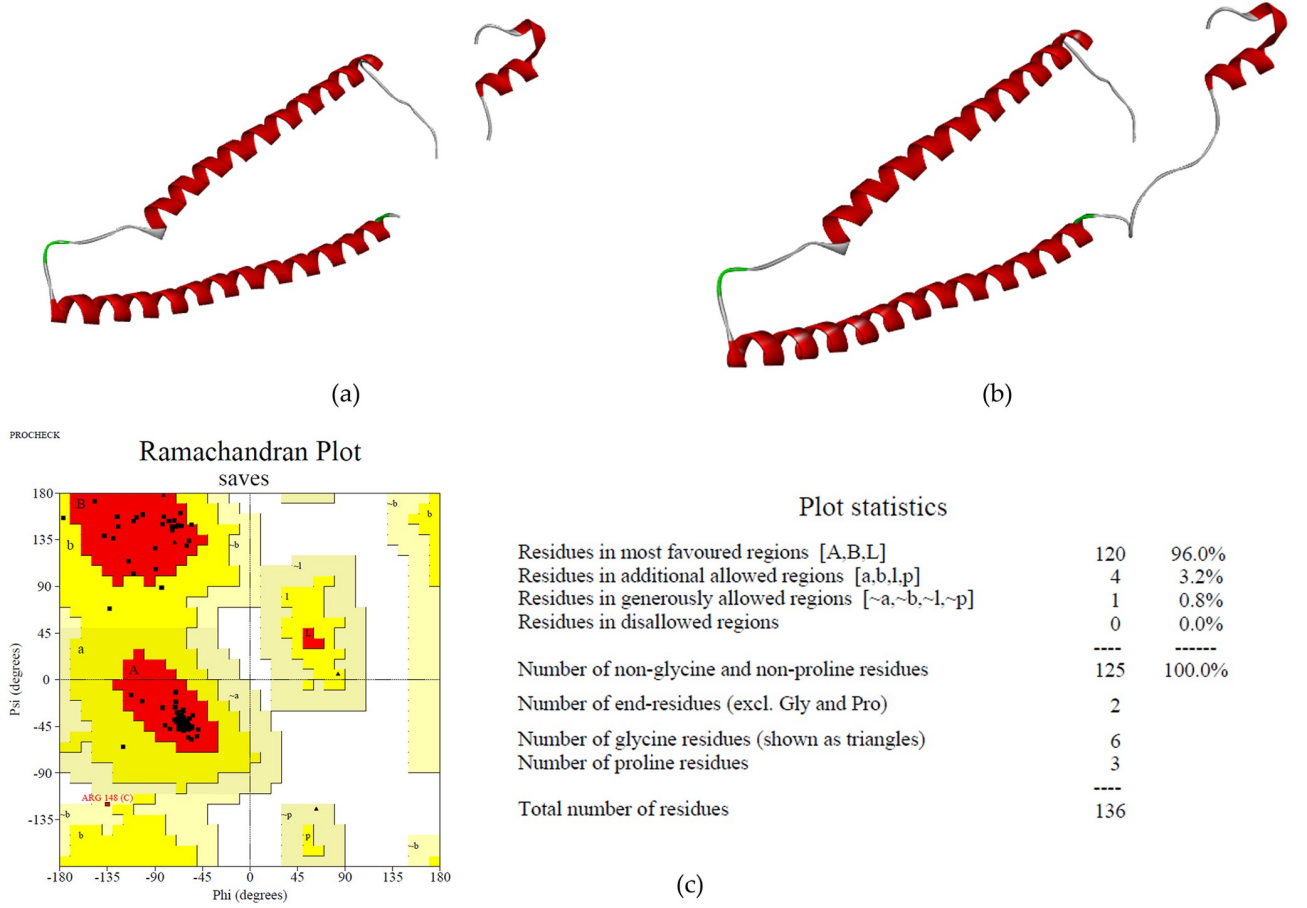
cTnI structure obtained from the RCSB website (a), cTnI structure after refining using PyMOL (b), and cTnI structure assessment (c).

Bioreceptor of Cardiac Troponin I (cTnI) was built by remodeling using a template from the RCSB website with the code 4Y99. The structure from the website comprises 166 amino acids. However, there are missing residues along Lys139-Val147 sequences compared to the original full sequence of the protein (Fig 1a). Then, we tried to refine the structure using PyMOL to make the structure closer to the real system. After refining the process using PyMOL software, we validated the structure using the SAVESv6.0 web server to examine cTnI quality (Fig 1b). ERRAT result showed a value of 87.395, and the Ramachandran Plot was 96% amino acids in the most favored regions, 4% amino acids in the additional allowed regions, and no amino acids in the prohibited regions (Fig 1c).

**Fig 2:**
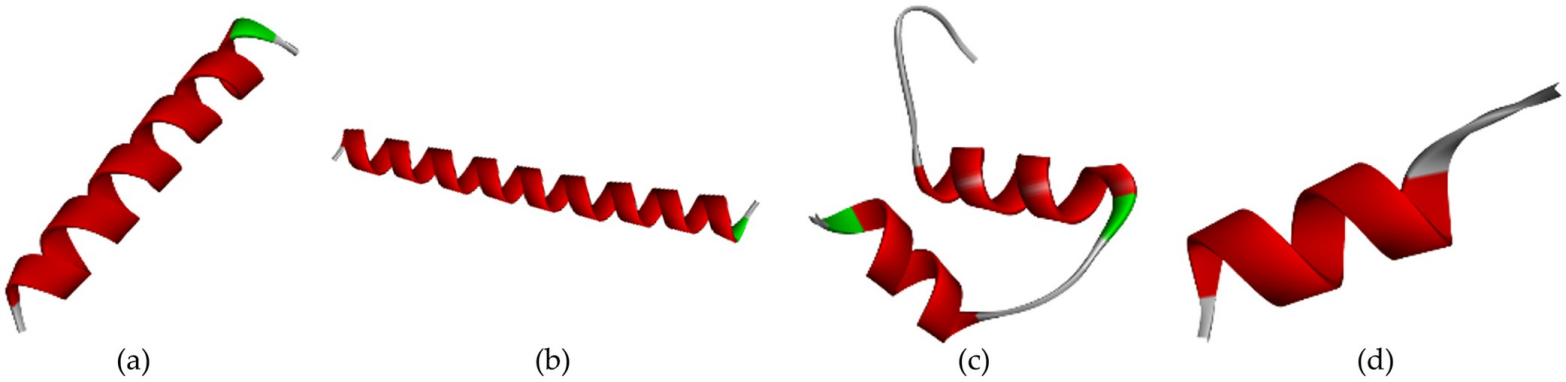
Peptide candidate structure modeling using the trRosetta server: (a) 3D structure of Peptide A with REKAKELWQTIYNLEAEKFDLQ sequence, (b) 3D structure of Peptide B with WQTIYNLEAEKFDLQEKFKQQKYEINVLRNRINDN sequence, (c) 3D structure of Peptide C with KNADGYIDLEELKIMLQATGETITEDDIEELMKD sequence, and 3D structure of Peptide D with FYSHSFHENWPS sequence.

The biomarker structure as our biosensor candidate was modeled using a template peptide in the RCSB web server that interacts with the cTnI protein (RCSB code 4Y99). To analyze the binding site between peptide and cTnI, we used the FTsite web server and previous research analysis [18]. From our analysis, we obtained three sequences that have interactions less than 4Å. Furthermore, we also used a sequence peptide from a previous research wet lab in the literature on the same target (FYSHSFHENWPS) [13]. To make a 3D model of these sequence peptides, we used the trRosetta webserver (Fig 2). According to Fig 2, the Peptide candidate was modeled and formed the secondary structure as mostly forming alpha-helix structure. After obtaining all the sequences, we continued to examine these sequences using the SAVES6.0 web server (Table 1). Table 1 shows the peptide candidate model evaluation using the SAVES v6.0 web server. The result revealed all peptides had good structural properties according to the Ramachandran plot.

**Table 1:** ERRAT and Ramachandran evaluation of peptide candidate.

Peptide sequence	ERRAT score	Ramachandran plot region			
		Core	Allowed	Generic	Disallowed
REKAKELWQTIYNLEAEKFDLQ (Peptide A)	100	100%	0%	0%	0%
WQTIYNLEAEKFDLQEKFKQQKYEINVLRNRINDN (Peptide B)	100	100%	0%	0%	0%
KNADGYIDLEELKIMLQATGETITEDDIEELMKD (Peptide C)	100	100%	0%	0%	0%
FYSHSFHENWPS (Peptide D)*	100	100%	0%	0%	0%

*literature peptide

### Molecular docking of candidate peptides

After building peptides as our biomarker candidate, we continued to assess their capability to build an interaction with the cTnI protein through the molecular docking process. This process was also used to select the best candidate peptide with the strongest interaction that can be processed further. To conduct molecular docking, we used ClusPro2.0 and HADDOCK 2.4 web servers. In addition, we also used the PRODIGY web server to obtain binding energy (ΔG) from the docking result. The result of the molecular docking is presented in Table 2. Based on the table. the second peptide or peptide B obtained the lowest affinity energy both in the ClusPro and HADDOCK compared to other peptides. To analyze further, we took a more detailed in the type of interaction of the peptide B with the cTnI protein. These interactions are presented in Table 3.

**Table 2:** Molecular docking result from the candidate peptides.

Peptide	ClusPro score	ΔG (kcal/mol)	HADDOCK score	ΔG (kcal/mol)
Peptide A	-2669.8	-7.8	-73.8 ± 6.4	-7.3
Peptide B	-3447.8	-10.4	-99.7 ± 10.4	-9.5
Peptide C	-2022.8	-9.1	-117.9 ± 7.7	-9.3
Peptide D	-2386.1	-6.7	-78.5 ± 4.0	-6.2

**Table 3:** Chemical interactions between the selected peptide and the cTnI protein.

Web server	Interaction		
	Type	Residue (peptide-cTnI)	Distance (Å)
ClusPro2.0	Hydrogen	GLU 24—ARG 68	2.74
		GLN 20—GLU 71	2.83
		ASP 13—LYS 72	2.73
		ASP 13—LYS 72	2.57
		GLN 21—LYS 72	2.71
		TYR 5—ALA 80	2.78
		GLN 15—VAL 107	2.96
		GLN 15—ASP 108	3.21
		ASN 26—VAL 118	3.16
		ASN 33—THR 129	2.73
		ASN 35—ARG 136	2.97
		ASN 35—ARG 136	2.66
	Salt bridge	GLU 24—ARG 68	2.74
		ASP 13—LYS 72	2.57
		GLU 16—ARG 79	2.81
		LYS 22—GLU 115	2.66
HADDOCK2.4	Hydrogen	ARG 31—GLU 64	2.7
		GLU 24—LYS 72	2.72
		GLU 24—LYS 72	2.6
		GLU 16—ARG 79	2.66
		TYR 5—LEU 83	3.26
		TYR 5—LEU 83	3.19
		GLN 15—VAL 104	2.82
		LYS 22—GLU 115	2.59
		ASN 26—THR 119	2.82
		ASN 33—THR 129	2.82
	Salt bridge	ARG 31—GLU 64	2.7
		GLU 24—LYS 72	2.6
		GLU 16—ARG 79	2.66
		LYS 22—GLU 115	2.59

**Fig 3:**
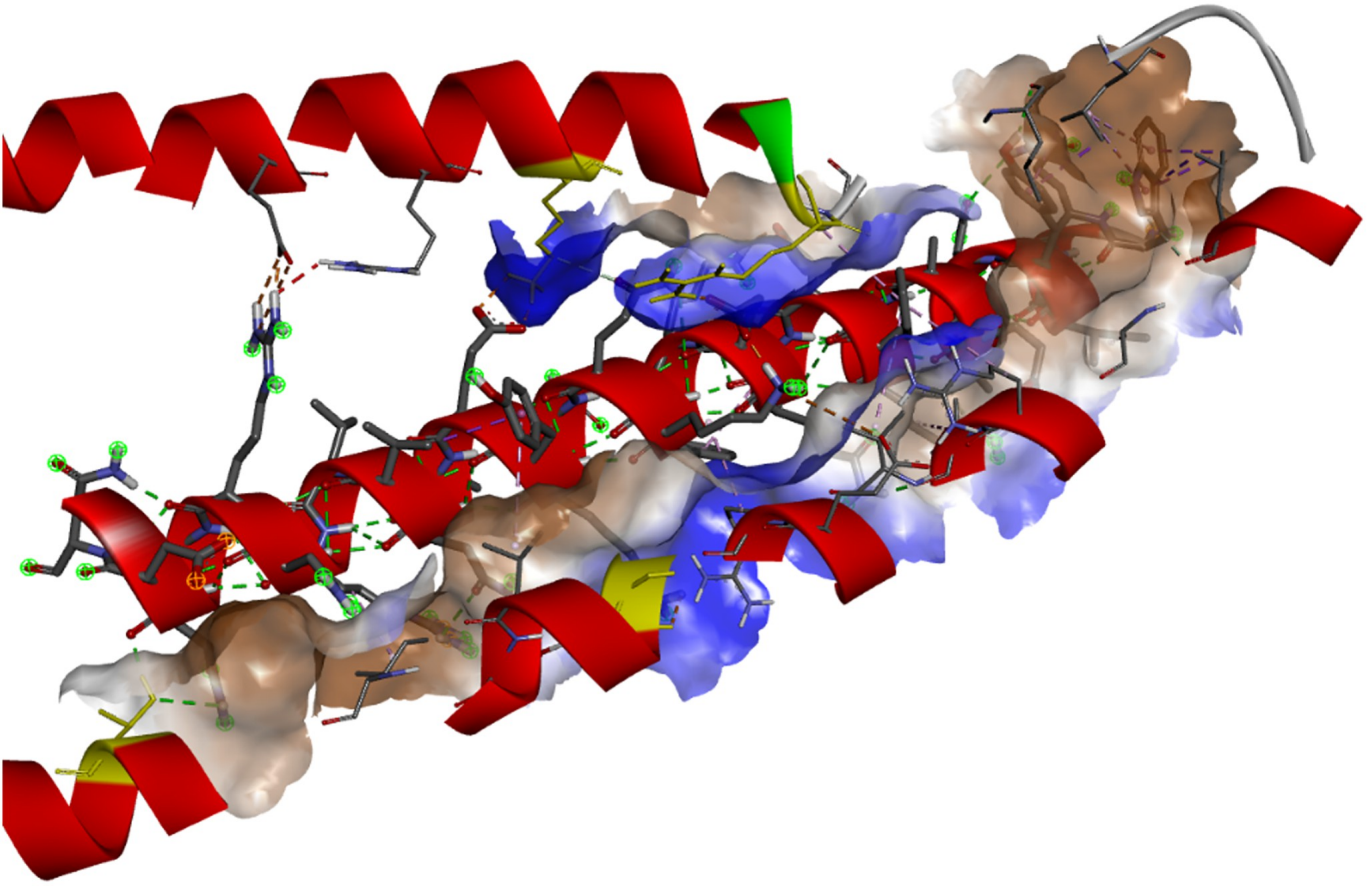
The hydrophobic pocket region between cTnI and peptide B interaction. The yellow part shows the residue for LYS72, ARG79, GLU115, and THR129. The middle peptide represents the Peptide B structure.

**Fig 4:**
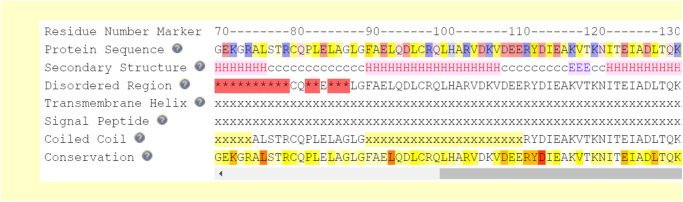
cTnI protein sequence characteristics. Residue LYS72, ARG79, GLU115, and THR129 represent K, R, E, and T respectively. Yellow to red means more conservative residue.

Based on the results from the ClusPro 2.0 server, 12 hydrogen bonds, 4 bonds salt bridges, and 179 non-bonded interactions have been built by the interaction between peptide B and the cTnI protein. Meanwhile, based on the results from the HADDOCK 2.4 server, there are 10 hydrogen bonds, 4 salt bridge bonds, and 169 non-bonded interactions (Table 3). The blue color of some cells in Table 3 indicated the most frequent residue involved in the interaction. Furthermore, Fig 3 reveals the hydrophobic region in the interaction. Specifically, some residues according to Table 3 that build hydrogen and salt bridge bonding are located in the hydrophobic pocket. Fig 4 shows the conservative residue from the cTnI protein. The more red color, the more conserved the residue.

### Biomarker point mutation and validation

We used the point mutation approach to improve the quality of peptide B as our chosen biomarker candidate. This method changes at least one amino acid from the sequence of peptide B. To conduct this, we used the BeAtMuSiC webserver. The result of the mutation server is a list of each original sequence and the amino acid recommendation. Then, we change the original peptide sequence based on the sequence mutation from the web server result. The mutation peptide was docked to the cTnI protein similar to the method in the C2 section. The top 20 with the lowest binding affinity are displayed in Table 4.

**Table 4:** Top 20 binding affinities after one point mutation.

Peptide	ClusPro score	ΔG (kcal/mol)	HADDOCK score	ΔG (kcal/mol)	RMSD
L14Q	-3456.9	-13	-96.6 +/- 19.6	-13.2	1.4 +/- 0.9
K11W	-3534.5	-12.5	-117.7 +/- 17.7	-12.9	1.1 +/- 0.7
L14D	-3322.1	-12.8	-88.9 +/- 20.5	-12.6	1.8 +/- 1.1
K19L	-3477.3	-11.6	-105.3 +/- 21.1	-12.6	2.0 +/- 1.2
I25W	-3552.1	-11.5	-116.4 +/- 35.3	-12.5	1.4 +/- 0.9
E8I	-3363.3	-11.6	-108.5 +/- 17.2	-12.3	1.3 +/- 0.8
Q21M	-3361.9	-11.9	-90.7 +/- 10.7	-11.8	1.7 +/- 1.0
K19Y	-3686	-11.6	-79.8 +/- 6.7	-12.1	6.8 +/- 0.3
L28E	-3405	-11.2	-100.5 +/- 7.8	-12.4	2.7 +/- 0.7
N30P	-3331.6	-11.1	-73.6 +/- 16.5	-12.4	3.1 +/- 0.2
K11F	-3554.3	-11.7	-100.2 +/- 14.7	-11.6	1.5 +/- 1.0
K19V	-3506.6	-11.3	-86.0 +/- 16.6	-11.8	3.8 +/- 0.3
K11Y	-3566.6	-12.3	-96.5 +/- 10.6	-10.2	2.9 +/- 0.6
K22L	-3629.9	-11	-85.2 +/- 17.2	-11.4	4.4 +/- 0.7
L14R	-3541.6	-11.6	-102.5 +/- 13.6	-10.7	1.5 +/- 0.9
E8W	-3846.1	-12.3	-127.4 +/- 10.5	-9.8	1.6 +/- 0.9
E8F	-3513.1	-11.6	-110.5 +/- 11.6	-10.4	3.6 +/- 0.3
K19F	-3485	-12.3	-103.5 +/- 22.1	-9.5	1.4 +/- 1.0
L14E	-3283.4	-9.7	-89.5 +/- 19.5	-9.2	1.8 +/- 1.1
K19W	-3563	-9.9	-98.6 +/- 2.6	-8.6	4.7 +/- 0.1

The 20 best point mutations as shown in Table 4 have resulted from only one mutation in the sequence. Then, we continue to combine the point mutation into two and three combination mutations based on the Table 4 result and assess the binding affinity of each mutated sequence. Tables 5 and 6 show the top 10 and 3 of the next mutation result.

**Table 5:** Top 10 binding affinities after two-point mutations.

Peptide	ClusPro score	ΔG (kcal/mol)	HADDOCK score	ΔG (kcal/mol)	RMSD
E8W_L28E	-3910.9	-12.3	-138.3 +/- 8.7	-14.1	1.6 +/- 0.9
E8W_K19V	-3922.2	-15.3	-108.3 +/- 6.7	-9.5	2.7 +/- 0.4
E8W_K11W	-3830.8	-11.9	-99.0 +/- 17.9	-12	4.5 +/- 0.2
E8I_K19W	-3449.8	-13.2	-101.5 +/- 14.6	-9.4	1.8 +/- 1.0
E8W_K11Y	-3671.6	-10.5	-105.8 +/- 14.7	-11.8	4.0 +/- 0.3
E8L_L14E	-3455.9	-10.6	-76.9 +/- 7.5	-11.6	3.4 +/- 0.2
E8I_L14Q	-3407.6	-12.5	-93.7 +/- 12.0	-9.2	14.1 +/- 0.1
E8W_K19Y	-3877.9	-11.7	-100.6 +/- 10.7	-9.7	5.0 +/- 0.6
E8F_K19W	-3813.5	-12	-104.9 +/- 14.1	-8.8	1.3 +/- 0.9
E8F_K19Y	-3782.4	-10.3	-106.6 +/- 9.7	-8	4.2 +/- 0.4

**Table 6:** Top 3 binding affinities after three-point mutations.

Peptide	ClusPro score	ΔG (kcal/mol)	HADDOCK score	ΔG (kcal/mol)	RMSD
E8W_K19V_N30P	-3694.2	-16.1	-129.8 +/- 8.9	-12.5	1.1 +/- 0.8
E8F_K19Y_L14E	-3796.6	-15.7	-120.4 +/- 11.1	-10.6	3.4 +/- 0.1
E8F_K19W_L14E	-3844.7	-13.2	-131.1 +/- 16.2	-11.9	1.7 +/- 1.0

According to Tables 5–7, we choose the top three peptide sequences that have the best binding affinity to continue to the molecular dynamic simulation. The selected peptides are shown in Table 7.

**Table 7:** Top 3 binding affinities after three-point mutations.

Peptide	ClusPro score	ΔG (kcal/mol)	HADDOCK score	ΔG (kcal/mol)	Nomenclature
E8W_K19V_N30P	-3694.2	-16.1	-129.8 +/- 8.9	-12.5	System 1
E8F_K19Y_L14E	-3796.6	-15.7	-120.4 +/- 11.1	-10.6	System 2
E8W_L28E	-3910.9	-12.3	-138.3 +/- 8.7	-14.1	System 3
Literature peptide	-2386.1	-6.7	-78.5 ± 4.0	-6.2	System 4

### Molecular dynamics simulation

The molecular dynamics simulation was done for systems 1, 2, 3, literature peptide, and cTnI-only protein (System 0). The literature peptide was used to make a comparison between the wet-lab peptide and our computational peptide design. The result of the molecular dynamics simulation was presented by RMSD (Root Mean Square Deviation), RMSF (Root Mean Square Fluctuation), SASA (Solvent Accessible Surface Area), RG (Radius of Gyration), and hydrogen-bond during the simulation process.

**Fig 5:**
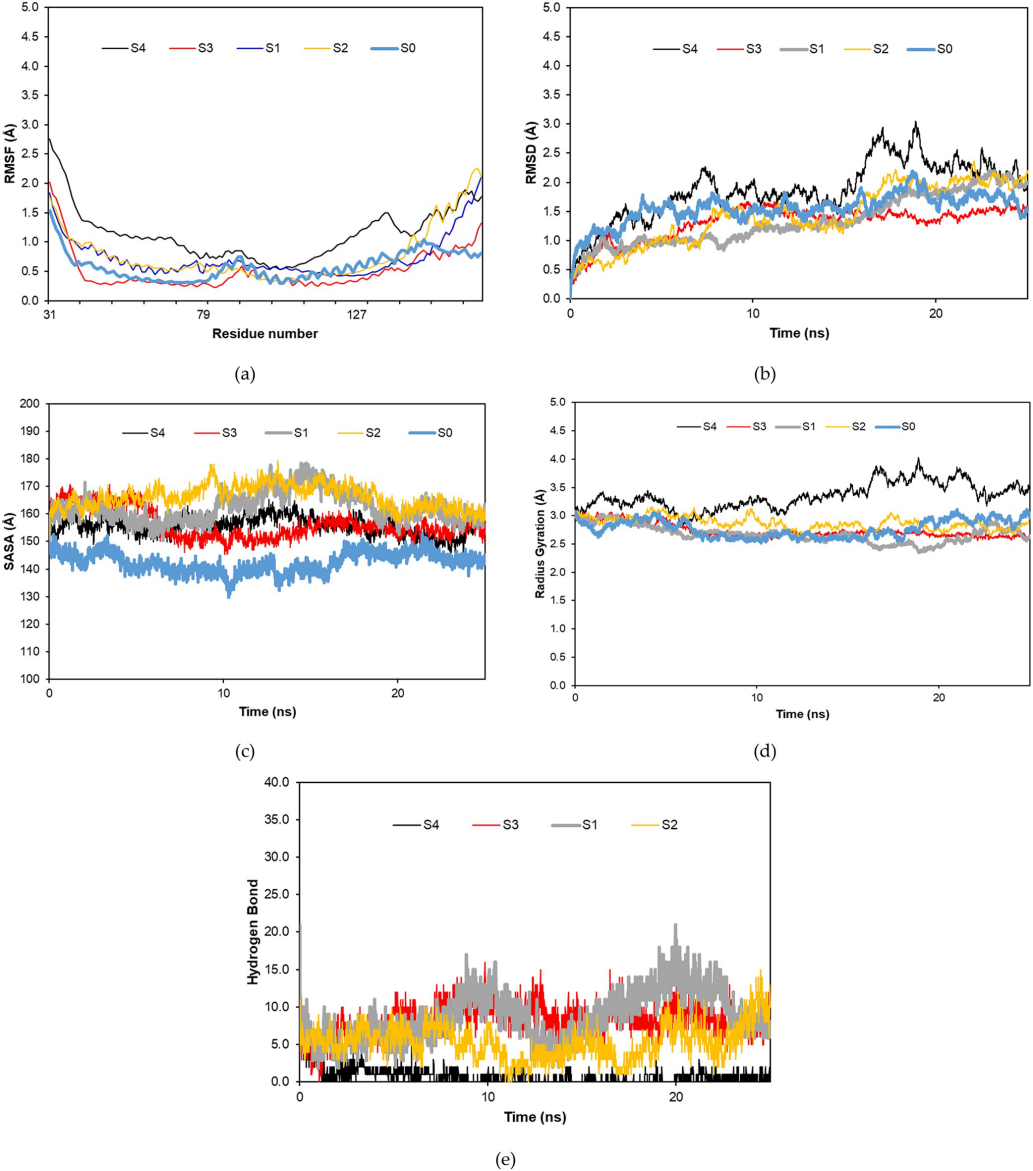
Molecular dynamic result RMSF (a), RMSD (b), SASA (c), RG (d), and hydrogen bond (e) during 25 ns.

Fig 5 shows the molecular dynamics simulation of all systems during 25 ns. In the RMSF result (Fig 5a), system 3 shows the lowest fluctuation during the simulation. Conversely, system 4 becomes the most fluctuating simulation of other systems. Moreover, almost all systems obtain their stability after 10 ns in the RMSD result (Fig 5b). System 3 shows the best stable RMSD, although it still has some fluctuations that are slightly higher than systems 1 and 2. In the RMSD result, system 1 also shows the most fluctuating system. Furthermore, from the RMSF and RMSD results, system 0 as a cTnI-only protein shows no significant difference to systems 1, 2, and 3. On the other side, system 0 has the lowest SASA result than other systems, and it is similar to the RG result (Fig 5c and 5d). Meanwhile, in the hydrogen bond during the simulation, systems 1 and 3 have the most hydrogen bonds (Fig 5e).

Table 8 shows the average value of the molecular dynamics result of each system during 25 ns. According to the table, system 3 becomes the best system among the results. Therefore, system 3 will be assessed further in the next assessment.

**Table 8:** Top 3 binding affinities after three-point mutations.

Name	RMSF	RMSD	SASA	RG	Hbond
System 1	0.782 ± 0.239	1.433 ± 0.506	166.265 ± 4.234	2.875 ± 0.107	8.688 ± 3.225
System 2	0.731 ± 0.351	1.340 ± 0.435	162.509 ± 5.401	2.677 ± 0.150	5.340 ± 2.288
System 3	0.509 ± 0.338	1.309 ± 0.293	156.221 ± 5.278	2.735 ± 0.116	8.320 ± 2.191
System 4	1.146 ± 0.444	1.878 ± 0.493	155.662 ± 3.281	3.349 ± 0.231	0.780 ± 1.007
System 0	0.588 ± 0.239	1.553 ± 0.260	142.560 ± 3.726	2.771 ± 0.153	-

### Specific interaction assessment

#### Protein receptor preparation

**Fig 6:**
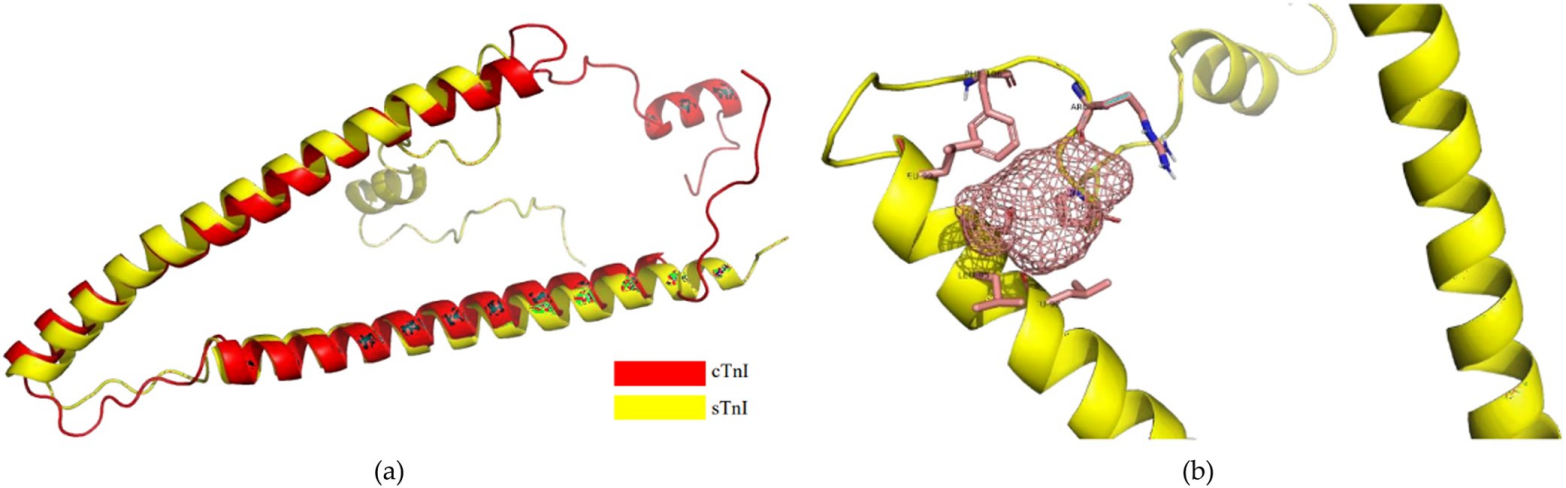
cTnI and sTnI structure comparison (a) and sTnI interaction site (b).

False positive has to be avoided regarding the development of biosensors. Hence, to ensure our biorecognition peptide is targeting the cTnI protein, we examine the interaction between system 3 to the sTnI (skeletal troponin I) protein (see Fig 6). sTnI protein is also released to the bloodstream when myofilament is damaged and has a similar structure to cTnI protein. Therefore, the existence of the sTnI protein can disturb our peptide-cTnI interaction and lead to a false positive result.

#### Molecular docking with sTnI protein

To examine and compare the interaction between system 3 to the sTnI protein, molecular docking is conducted. The interaction between system 3 to the sTnI protein was named as system 5 and the comparison result is shown in Table 9.

**Table 9:** Molecular docking comparison between cTnI and sTnI to system 3.

System	HADDOCK score	ΔG (kcal/mol)
System 3	-138.3 +/- 8.7	-14.1
System 5	-57.7 +/- 2.6	-7.3

**Fig 7:**
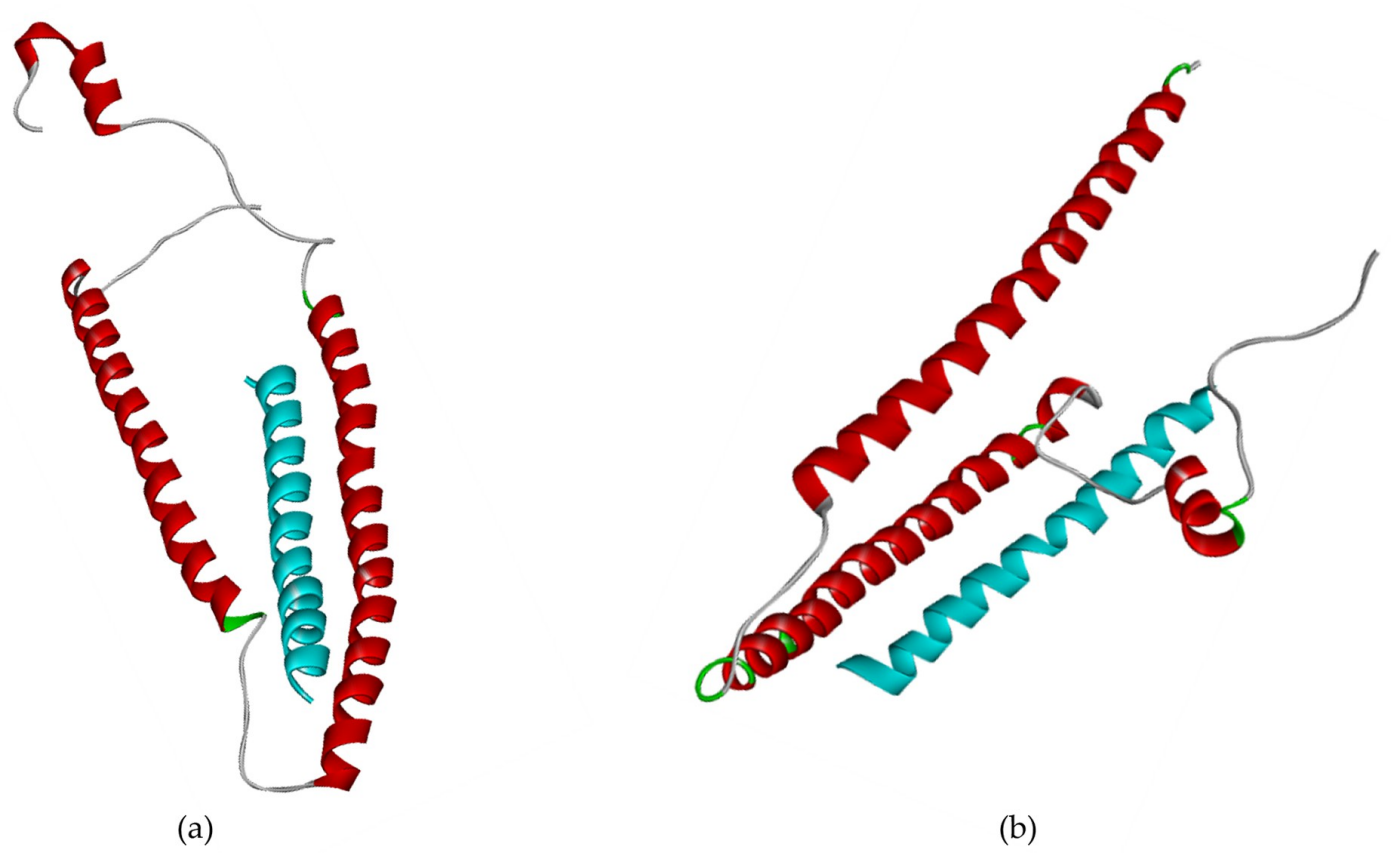
System 3 (blue) complex with cTnI (red) (a) and sTnI proteins as system 5 (red) (b).

Table 9 shows that System 3 has better interaction than System 5. Either the HADDOCK score or binding affinity, system 3 obtains around two times higher value in the interaction with cTnI protein than system 5 with sTnI protein. Furthermore, Fig 7 shows the different poses from system 3 to build the interaction with cTnI and sTnI proteins.

#### Molecular dynamics simulation with sTnI protein

**Fig 8:**
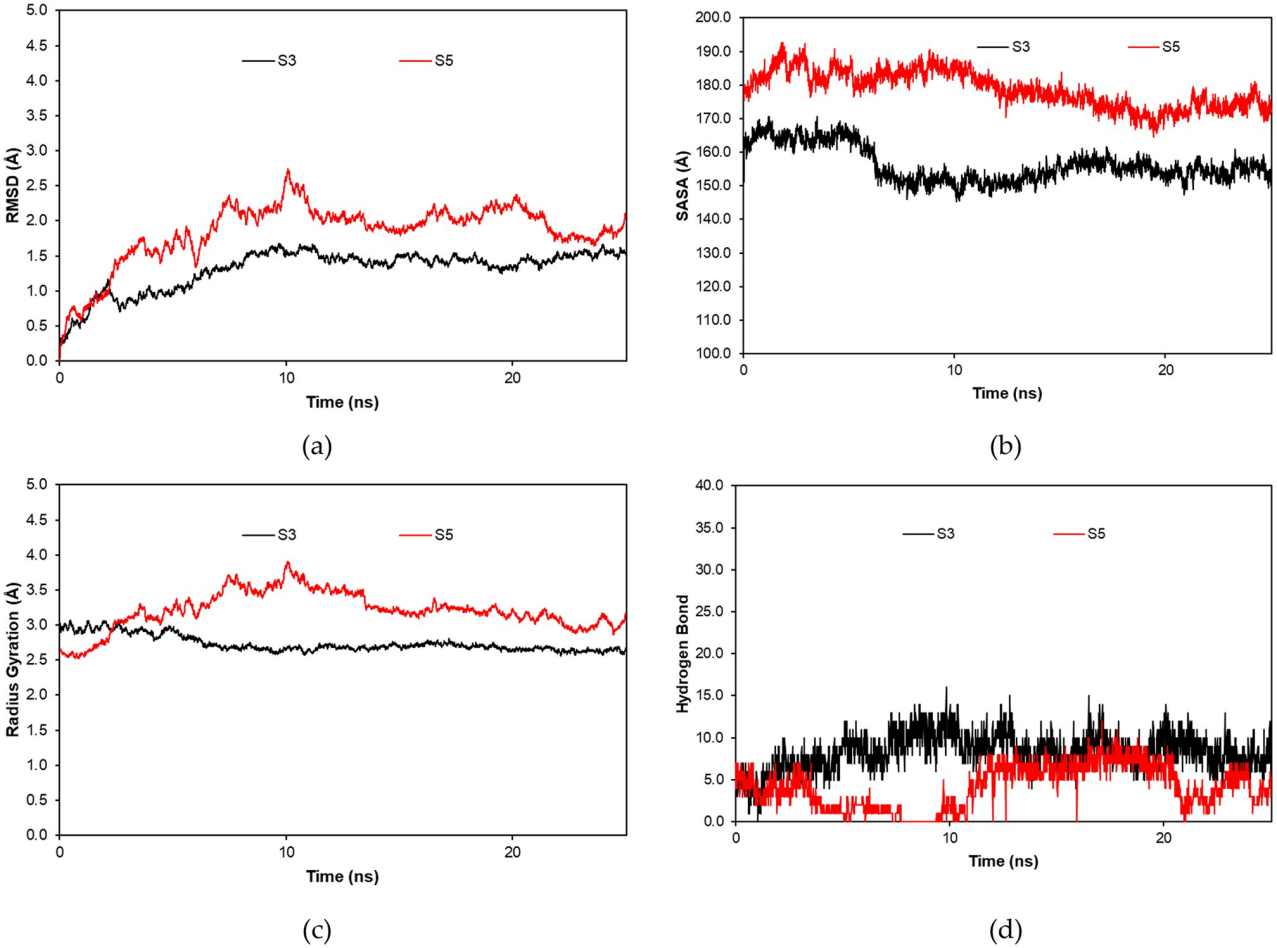
Molecular dynamics result of RMSD (a), SASA (b), RG (c), and hydrogen bond (d) between systems 3 and 5 during 25 ns.

According to Fig 8, it is clear that System 3 has better stability than System 5. System 5 shows more fluctuation, implying the interaction between the biorecognition peptide to the sTnI protein is not stable. Moreover, the most noticeable result is the absence of hydrogen bonds at certain times in System 5.

## Discussion

Our study reveals the computational method of the biorecognition peptide as a cardiovascular biosensor design. Cardiac troponin is a contractile protein that commonly has three forms: cTnC, cTnI, and cTnT, with their sizes 18, 23, and 35 kD, respectively. Although they are involved in a similar role as a regulator in the contraction and relaxation of the myocardium, they have different specific functions. cTnC works for the Ca2+ binding subunit, cTnI inhibits actin-myosin contraction, and last, cTnT works for binding troponin to tropomyosin [21]. The three forms of cardiac troponin can be detected in the bloodstream. However, cTnI is more specific for cardiac failure detection. This characteristic leads to its usefulness not only in standing as the gold standard method for acute coronary syndrome detection but also in classifying myocardial infarction into some levels [22, 23].

In our research to develop cTnI peptide-based biosensors, the cTnI protein must be modeled. To develop cTnI protein structure we obtain the template from the RCSB website with a code 4Y99. 4Y99 is a template comprising three cardiac structures: cTnT, cTnC, and cTnI. cTnI protein structure is refined from the template and the result from refining is assessed using some quality assessments of the protein. From the quality assessment using the web server, our cTnI structure reveals a good result. The non-bonded interaction between different atoms was analyzed by ERRAT, and the result is 87.395, representing a good quality model. Furthermore, based on the Ramachandran Plot, there are no amino acids located in the prohibited region, with 96% of amino acids in the most favored region representing that this model has a high stereochemical quality (Fig 1).

The next step in designing a cTnI biosensor by building a sequence peptide that can interact with the cTnI protein. Some previous studies determined the binding site between the peptide and protein receptor by using a template peptide-protein interaction in the RCSB web server [9, 10, 24, 25]. In our study, we use the interaction between cTnC and cTnT to the cTnI protein, as provided in the crystal structure of 4Y99, to determine the active site of the cTnI protein, which is also supported by the research literature. Furthermore, to gain more accuracy, we use the FTsite web server analysis to determine the active site of the cTnI protein. To determine which sequences are used, the distance interaction less than 4 Åbecomes a cut-off. Tam et al [26] and Desantis [27] stated that a distance less than 5 Å between two amino acid centroids is used as a standard measurement for possible interactions. Furthermore, Spassov [28] showed a distance of less than 4 Å can build a stable salt-bridge interaction.

We design three peptide sequences as a biosensor candidate. Moreover, Park [13] have developed high-affinity peptides for the recognition of the cTnI protein, therefore we also use the sequence as a comparison peptide from the wet-lab result. Four sequence peptides are then built in the 3D model using the trRosetta web server. The lengths of the peptides are 22, 34, 34, and 12, respectively (Fig 2). Pavan [29] gives a review that deals with short peptides (up to 50 amino acids) as biomimetic active recognition elements in sensing systems. Short peptide reveals a good capability for the biorecognition design because of its fast-screening peptide libraries using a computational method, low cost, easy modeling, and ease of modification to enhance binding activity further. Furthermore, based on Fig 2, the secondary structure of the designed peptide was mostly formed as an *α*-helix structure. Regarding the protein activity, various protein-protein and protein-DNA interactions were also influenced by their secondary structure. *α*-helix is the common secondary structure that involves the protein-protein and protein-DNA interactions responsible for maintaining complex biological activities. Furthermore, a small peptide with an *α*-helix structure is a promising form in the medical field [30]. However, the peptide must be modified to enhance its desired activity. For instance, extended random-coil conformations are more susceptible to proteolysis activation if the peptide is designed for human body systemic activity.

Research conducted by Wang and Feng [31] investigated how certain side chain structures affect amino acid residue’s tendency to adopt an *α*-helix conformation. The study revealed that particular amino acid residues tend to form hydrogen bonds with neighboring residues through either the main chain-to-side chain or side chain-to-side chain interactions. This suggests that there might be a preference for specific residues to interact with neighboring residues depending on their position within the *α*-helix. Wang and Feng [31] stated that ALA, ARG, GLN, GLU, ILE, LEU, LYS, MET, PHE, SER, TRP, and TYR are amino acids that have individual strong or medium to tend to form alpha-helix structures within a peptide. According to Fig 2, Peptide C was mixed with *α*-helix and loop structure, while Peptides A and B were dominant with *α*-helix structure. It was in line with the amino acids stated by Penel et al. (1999) that Peptides A and B contained more for the type of amino acids that tend to form *α*-helix structure.

After building these peptides, they are further analyzed for their structure quality. Overall, all peptides are of excellent quality based on Table 1 from ERRAT and Ramachandran Plot results. The Ramachandran plot displays the phi-psi torsion angles for every residue within the structure. Glycine residues are excluded from the assessment since it does not adhere to the areas of the plot suited for other sidechain types. Ideally, aiming for more than 90% of residues to fall within core regions would be desirable. The proportion of residues situated in the core areas serves as a reliable indicator of stereochemical integrity. Furthermore, there was no residue plotted in the disallowed region based on Table 2, hence the quality of the peptide was good enough to be used further as peptide structure.

To continue designing the best peptide biosensor, molecular docking is conducted among these peptides to examine the interaction capability with the cTnI protein. In this research, we employed ClusPro and HADDOCK for molecular docking. This was conducted to ensure the docking result was the best, even using different docking algorithms despite these being suitable for studying protein-protein interaction. Previous research already used ClusPro and HADDOCK to validate their docking results. Rajendaran’s team [32] used ClusPro and HADDOCK to dock jacalin into the receptor binding domain (RBD) of SARS-CoV2. According to the result, the docking score from ClusPro and HADDOCK showed a similar trend. Ghobadi and coworkers [33] conducted docking of a new peptide design to the HLA-A*03 complex to prevent multiple sclerosis. The method was done to validate the interaction mode of each model and the result also showed a similar trend with the HADDOCK result as the main reference. Furthermore, Shang [34] performed two docking screenings using ClusPro and HADDOCK, respectively. Although they use these servers for first and second screening docking, however, the concept for using different docking algorithm approaches to ensure the best result was similar to ours and other previous research.

Table 2 shows that Peptide B has the lowest binding energy based on the result of two web servers than other peptides. Based on Table 3, either using ClusPro or HADDOCK web servers reveals an almost similar quantity of Peptide B’s hydrogen and salt bridge interaction. Hydrogen bond and salt bridge interaction are considered to be a strong interaction in protein chemistry and have an essential role in the interaction between protein and peptide. Among protein interactions, hydrogen bonds play a major role in the recognition of small peptides by a protein generating an initial interaction [35]. The interaction can also stabilize protein complexes through the interaction with hydrophilic sidechains that have a high charge density. Moreover, hydrogen bonding is also utilized to minimize the entropic cost of transitioning from a highly flexible, unstructured peptide to a well-defined rigid structure in a complex with protein [35]. Although the salt bridge bonding is not as much as hydrogen interaction in the Peptide B-cTnI complex, the salt bridge also has an important influence on the complex. A salt bridge is a chemical bonding between the base of an alkaline amino acid, which is in the protonated form, and an acidic amino acid, which is in the deprotonated form. However, GLU amino acid can form either neutral or protonated form as it is a weak alkaline amino acid, although GLU does not appear in our result (Table 3) [36]. Furthermore, in some cases, salt bridge interaction can be stronger, such as when the interaction occurs in the hydrophobic pockets [36].

According to Table 3, LYS72, ARG79, GLU115, and THR129 become the cTnI residue that is most frequently involved in the interaction with Peptide B either using ClusPro or HADDOCK web server. All of these residues almost build an interaction in the hydrophobic region (Fig 3). In some previous protein-peptide interaction research, a hydrophobic pocket is important as a binding site for stabilizing the interaction of these molecules [36–39]. Xie [36] and Kaufmann [39] stated that hydrophobic pockets could enhance the strength of hydrogen and salt bridge interactions. The significance of hydrophobicity lies in its role in facilitating protein-protein interactions and the binding of various molecules. Typically, these interactions occur in groups, with the binding sites predominantly situated within the most robust hydrophobic clusters on the surface of the protein acceptor. Additionally, a study has indicated that the most hydrophobic cluster aligns with over one-third of the surface area covered by the bound ligand [40].

To look for more details, a conserved residue analysis is conducted (Fig 4). Frieden [41] stated that conserved amino acids in the structural protein are related to the protein function. Moreover, Ma [42] and Guharoy and Chakrabarti [43] show that conserved amino acids can distinguish between functionally important residues, including catalytic activity or binding, or are responsible for providing stability to the folded structure and for exposed protein surfaces. Fig 4 shows LYS72 is the most conserved amino acid compared to ARG79, GLU115, and THR129, which has a more red color. Moreover, from the figure, the conserved residues are near each other. Guharoy and Chakrabarti explained conserved residues tend to form a cluster to support each other functions. However, some proteins also show more than one cluster of conserved amino acids for a larger structure, such as an enzyme [43]. These multiple clusters of conserved regions represent the more complex of the protein functions. The multiple clusters may be important for stabilizing the interaction in the case of larger interfaces by forming distinct binding units.

After analyzing the molecular docking result, the best peptide or Peptide B is subjected to the point mutation process. In the previous research, there were some approaches to enhance the peptide structure and function. Xiao [10] showed cTnI biosensor peptides development using an evolution-based algorithm that was built initially using the Amber force field 99SB. Next, Baig [24] developed peptides for targeting the SARS-CoV-2 spike using alanine scanning by changing each peptide’s residue using alanine and assessing the binding result after changing. Furthermore, Badhe [11] also developed peptides for SARS-CoV-2 using a list of peptides from previous research and selecting them according to the molecular dynamic simulation result. In the next year, Mastouri [9] developed peptide biosensors for detecting interleukin-6 by random mutation approach. In our research, sequential point mutation is used to optimize the mutation result. Point mutations can significantly influence the thermodynamic stability of proteins, potentially affecting the protein’s function. Consequently, a common objective in biotechnology is to intentionally enhance or maintain stability while modifying specific protein properties. This goal is often pursued in various fields, such as optimizing industrial processes, drug design, and basic research [44]. Furthermore, there are some general principles responsible for protein-protein binding interaction, such as hydrophobic contacts and electrostatic interactions. These residues are referred to as ‘hotspots’, and these are generally subjected to the mutation design to enhance the protein’s functional ability. To perform the mutation process, we use the BeAtMuSiC web server that mutates a protein into any kind of amino acid and provides the binding affinity as the result of the mutation [20].

In the point mutation process, we perform sequentially mutation processes. The process is done from one amino acid mutation to two and three amino acid mutations based on the result of the one residue mutation (Tables 4–6). This approach is dedicated to gaining more datasets that have better binding affinity results as we choose random mutation during the mutation process. According to Tables 4–6, tryptophan (Trp) residue coded by W is the most common substituent amino acid suggested by the web server. Also, it contributes to the best sequence result (Table 7). Trp has the lowest abundance of amino acids compared to the other 20 amino acids, while leucine has the most abundance. However, despite its poor abundance, Trp has an important role in protein function and stability. This characteristic is gained by Trp’s unique structure. The nitrogen of its indole ring can act as a hydrogen bond donor also the ring is considered the strongest cation-p binder of all aromatic side chains. Furthermore, its hydrophobic chain can build extensive van der Waals interactions within protein hydrophobic cores [45]. As the result of sequentially mutation processes, we chose the best three mutated peptides that have the lowest binding affinity to the cTnI protein and also a peptide from the literature that had been developed in the wet lab (Table 7). These peptides will be analyzed further using molecular dynamic simulation.

**Fig 9:**
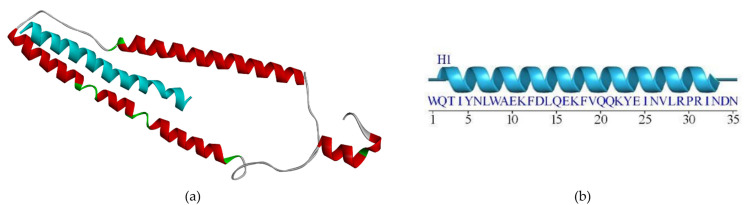
System 1 (blue) complex with cTnI (red) protein (a) and the sequence of system 1 (b).

**Fig 10:**
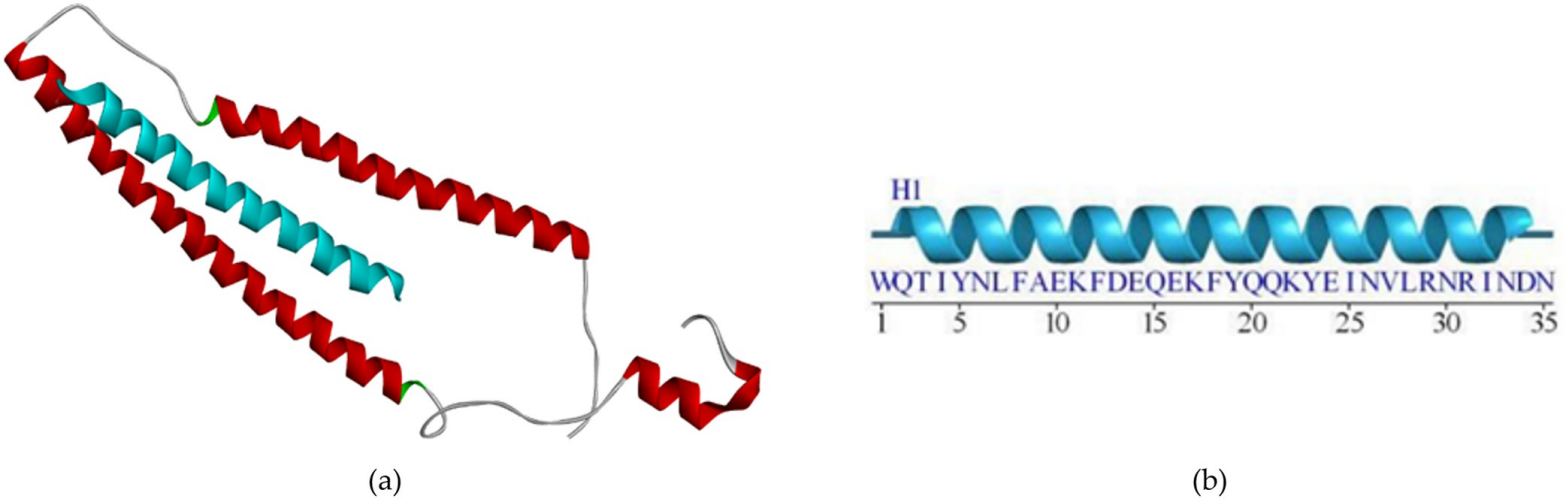
System 2 (blue) complex with cTnI (red) protein (a) and the sequence of system 2 (b).

**Fig 11:**
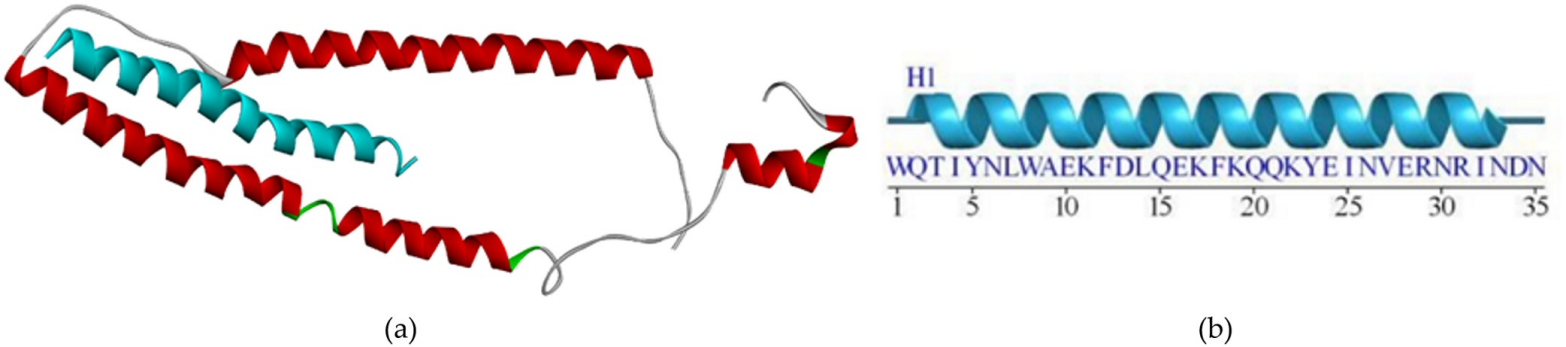
System 3 (blue) complex with cTnI (red) protein (a) and the sequence of system 3 (b).

Molecular dynamics is conducted to run the three best peptides referred to as System 1, 2, and 3 (Figs 9–11) also a literature peptide as System 4 and the cTnI structure only (System 0) for 25 ns. In contrast to molecular docking, molecular dynamics can show the flexibility of the ligand and receptor within a certain solvent, allowing the receptor to change its conformation [46]. The first assessment is the RMSF value to measure each amino acid residue in the entire protein in a certain period. Fig 5a reveals System 4 as the most fluctuating peptide compared to the others, while System 3 has the lowest fluctuating and is almost similar to System 0 (S0). System 0, as the cTnI-only protein, has stability in the C-terminal region. According to Sheng and Jin [18], that region is used for some functions such as cTnC and tropomyosin bindings, while the region around residue of 80 to 100 is the most fluctuating region that acts as cTnT binding. Regarding that, almost all systems show a fluctuating residue in their C-terminal region except for System 3, which is similar to System 0. Interestingly, System 3 has a less fluctuating residue, around 80 to 100 compared to System 0. It may come from the cTnI-Peptide B interaction and shows that the peptide can stabilize the cTnI protein using their strong interactions.

Next, Fig 5b shows the RMSD result of all systems. RMSD indicates the atomic fluctuations in the entire protein at a certain time to examine the changing conformation to its reference structure. System 3 becomes the most stable complex during 25 ns in the RMSD result. This result is correlatively positive with the SASA and Radius of Gyration (RG) results as shown in Fig 5c and 5d. RG assesses the compactness of the protein structure, while SASA means the area of the protein that is exposed to the environment. System 3 shows the stable atomic fluctuation from the RMSD result, then consequently its complex should be more compact and less area exposed to the outer condition without any significant changing formation in the complex leading to the less fluctuating result of SASA and RG. This compactness structure between System 3 with cTnI may come from hydrogen bonding involvement. Fig 5e shows that S3 has the most hydrogen bonds compared to the other systems with a stable interaction during 25 ns. Hydrogen bond plays a significant role in stabilizing protein complex interaction hence, taking consideration of this interaction is important [47]. Hydrogen bonding can involve many mechanisms such as specificity of binding, transport, adsorption, distribution, metabolization, and excretion characteristics of small molecules [48]. Also, hydrogen bonding promotes a higher binding affinity that leads to strong, complex interaction and reduces their fluctuation residues. This high affinity by hydrogen interaction is due to the reduced competitive interference with water [49].

According to the molecular dynamics results, System 3 becomes the best peptide to continue our assessment as a biosensor candidate for the cTnI protein. In the next step, the sTnI protein is used during the selectivity assessment. In clinical measurement, a false-positive result sometimes occurs for detecting myocardial infarction failure. In some cases, there is a cross-reaction of anti-cTnI and anti-cTnT antibodies with skeletal troponin molecules such as the sTnI protein. This happens when there is damage to the striated skeletal muscle, leading to some diseases such as myopathies and rhabdomyolysis. This cross-reaction commonly occurs in the first and second generation of troponin immunoassays because of their less specific antibodies’ interaction with the cardiac troponin [12]. Therefore, an assessment of the enhancing the specificity of the biosensor agent to the cTnI protein is important, especially, for clinical practice. Since the false decision may make incorrect diagnoses and treatment which can be dangerous to the patient.

Table 9 shows that the molecular docking result between Systems 3 and 5 differs quietly. System 3 is twice higher than System 5 in HADDOCK score and binding energy. This result is confirmed during the molecular dynamic simulation which System 3 shows a more stable complex and higher hydrogen bond interactions according to Fig 8. To look more detail from the hydrogen bond interaction, Table 10 shows that System 3 is stable in building hydrogen bonds during molecular dynamic simulation 25 ns. In contrast, System 5 failed to keep a constant interaction of hydrogen bonds. It may be a cause of making System 5 not as stable as System 3 according to the molecular dynamic result because hydrogen bond plays a vital role in the protein complex stabilization [47](Fig 8). Hence, System 3, as our biosensor candidate, is more sensitive to the cTnI protein and can build a stable interaction that is very crucial to build better biosensors for myocardial infarction.

**Table 10:** Interaction comparison between systems 3 and 5 during molecular dynamic simulation System Early simulation (t = 0 ns) Middle simulation (t = 12.5 ns) End simulation (t = 25 ns).

System	Early simulation (t = 0 ns)			Middle simulation (t = 12.5 ns)			End simulation (t = 25 ns)		
	Type	Residue	Non-bonded Interaction	Type	Residue	Non-bonded Interaction	Type	Residue	Non-bonded Interaction
System 3	Hydrogen interaction	ARG31—GLU 71	149	Hydrogen interaction	ARG29—GLU 62	101	Hydrogen interaction	LYS22—GLU66	110
		GLU28—LYS 72			ARG29—GLU 62			ASP13—SER77	
		GLU24—ARG 79			LYS22—GLU 66			ASN6—GLU84	
		GLU24—ARG 79			ASP13—SER 77			GLN2—LEU85	
		LYS17—ALA 80			GLN 2—LEU 85			GLN20—ARG103	
		TYR 5—GLU 84			ASN26—ARG111			LYS19—ASP108	
		GLN 2—LEU 88			ASN30—LYS117			TYR23—GLU110	
		LYS19—VAL104			ASN33—LYS117			ASN33—ASN121	
		ASN26—GLU115							
		ASN30—ASN121							
		ASP34—ASN121							
	Salt bridge	ARG31—GLU71		Salt bridge	ARG29—GLU 62		Salt bridge	LYS22—GLU66	
		GLU28—LYS72			LYS22—GLU 66			LYS19—ASP108	
		GLU24—ARG79			LYS19—ASP108				
					ASP34—LYS117				
System 5	Hydrogen interaction	TYR23—LYS87	81	Hydrogen interaction	GLU24—ARG69	53	Hydrogen interaction	GLU10—LYS105	26
		GLU24—LYS90			GLU24—ARG69			ASP13—LYS105	
		LYS19—GLU93			GLN2—GLU92				
		GLN20—ASP94			LYS22—GLU110				
		GLN20—GLN97							
		TYR5—LYS107							
		GLU16—ARG113							
		GLU16—ARG113							
	Salt bridge	GLU24—LYS90		Salt bridge	GLU24—ARG69		Salt bridge	GLU10—LYS105	
		LYS19—GLU93			GLU24—LYS72			ASP13—LYS105	
		GLU16—ARG113			ASP13—ARG79				
					LYS22—GLU110				

## Conclusion

To summarize our result, we have successfully developed a cTnI protein-based biosensor using a computational approach. Peptide B with the sequence “WQTIYNLWAEKFDLQEKFKQQKYEINVERNRINDN” becomes the best peptide that has the most stable and strong interaction with the cTnI protein. Furthermore, according to the selective test, peptide B is more stable and stronger for the cTnI protein compared to the sTnI protein. Therefore, our study provides a new potential candidate for myocardial infarction diagnosis to ease the burden of cardiovascular disease.
